# Association Between Risk Factors for Complications From COVID-19, Perceived Chances of Infection and Complications, and Protective Behavior in the US

**DOI:** 10.1001/jamanetworkopen.2021.3984

**Published:** 2021-03-31

**Authors:** Robert F. Schoeni, Emily E. Wiemers, Judith A. Seltzer, Kenneth M. Langa

**Affiliations:** 1 Institute for Social Research, University of Michigan, Ann Arbor; 2 Gerald R. Ford School of Public Policy, University of Michigan, Ann Arbor; 3 Department of Economics, University of Michigan, Ann Arbor; 4 Department of Public Administration and International Affairs, Syracuse University, Syracuse, New York; 5 Center for Aging and Policy Studies, Aging Studies Institute, Syracuse University, Syracuse, New York; 6 Department of Sociology, University of California, Los Angeles; 7 California Center for Population Research, University of California, Los Angeles; 8 School of Medicine, Department of Internal Medicine, University of Michigan, Ann Arbor; 9 Veterans Affairs Ann Arbor Center for Clinical Management Research, Ann Arbor, Michigan

## Abstract

**Importance:**

It is unknown whether adults who are susceptible to severe complications from COVID-19 recognize their susceptibility and modify behavior to reduce exposure.

**Objective:**

To determine whether adults with risk factors for COVID-19 complications perceive an elevated chance of complications and undertake fewer higher infection risk behaviors.

**Design, Setting, and Participants:**

This cross-sectional analysis, adjusted for sociodemographic characteristics, included civilian noninstitutionalized US adults of wave 18 of the Understanding America Study collected from November 11 to December 9, 2020.

**Exposures:**

US Centers for Disease Control and Prevention–identified medical risk factors for COVID-19 complications and older age.

**Main Outcomes and Measures:**

Primary outcomes were perceived percentage chance of infection and hospitalization and death if infected; whether 9 potentially higher infection risk activities were undertaken in the past week and, if so, whether a mask was worn; whether a mask was worn anywhere in the past week; and attitudes toward 12 aspects of mask wearing.

**Results:**

In Understanding America Study wave 18 (n = 5910 participants with nonmissing data), the mean age was 48 years, and 52% were women. The response rate was 77%. Adults with 7 of 9 medical risk factors and aged 70 years and older reported a higher perceived chance of complications if infected. Adjusted mean perceived chance of hospitalization if infected ranged from 23.9% (95% CI, 22.2%-25.5%) for those with high blood pressure to 40.4% (95% CI, 34.6%-46.2%) for those with chronic lung disease and was associated with number of medical risk factors: 17.6% (95% CI, 16.4%-18.8%) and 41.8% (95% CI, 38.7%-45.0%) for adults with 0 vs 3 or more medical risk factors, respectively. Fewer potentially higher infection risk activities were undertaken by adults with 3 or more vs 0 risk factors: 2.83 (95% CI, 2.66-2.99) vs 3.12 (95% CI, 3.02-3.22). Wearing a mask sometime last week was nearly universal (90.1%). But during only 1 specific activity (visiting a grocery store or pharmacy) did more than half always wear a mask, and for only 1 activity (visiting a grocery store or pharmacy) was mask wearing more common among adults with 3 or more vs 0 conditions.

**Conclusions and Relevance:**

In this cross-sectional survey study, adults with risk factors for COVID-19 complications reported higher perceived susceptibility to complications. During common activities, including visiting with friends, the majority of adults, including the highly susceptible, did not consistently wear masks.

## Key Points

**Question:**

Do adults with risk factors for COVID-19 complications understand their elevated risk and undertake fewer higher infection risk behaviors?

**Findings:**

In this survey study of 6084 US adults, those with 7 of 9 medical risk factors for COVID-19 complications reported higher perceived chance of hospitalization or death from COVID-19 if infected compared with those without the factor. While nearly all adults reported recent mask wearing, during several common activities the majority, including susceptible adults, did not consistently wear masks.

**Meaning:**

Adults with medical risk factors for COVID-19 complications understood their risks were higher, but consistent mask wearing remained low.

## Teaser

This cross-sectional survey study assesses whether adults with risk factors for COVID-19 complications perceive an elevated chance of complications and undertake fewer higher infection risk behaviors.

## Introduction

The US Centers for Disease Control and Prevention (CDC) provides information about who is at risk for severe complications from COVID-19 infection and what precautions to take to avoid infection.^1,2^ These public health notices assume that individuals will recognize that their underlying medical conditions make them susceptible and, because they are susceptible, will take steps to reduce their exposure to infection.

The assumption in these public health notices builds on a model of human behavior in which an individual’s perception of risk affects their decisions about protective behavior.^3^ Knowledge about health conditions predicts subjective probabilities of survival, and these survival expectations have been shown to predict actual mortality.^4^ The associations between perceptions of risk and protective behaviors are especially important in the case of emerging infectious diseases for which the actual risks and the efficacy of protective behaviors are uncertain.^5,6,7^ Evidence from early in the COVID-19 pandemic shows that individuals who think they are at greater risk of COVID-19 infection are more likely to take some actions to reduce their risk, such as handwashing, avoiding crowded places and high-risk persons, and postponing or canceling travel.^8^ Older age increases the perceived risk of dying from COVID-19 if infected.^9^ Evidence is lacking on whether chronic health conditions identified by the CDC as increasing the risks of severe complications are associated with individuals’ perceptions of risks or engaging in behaviors shown to reduce transmission, including physical distancing^10^ and mask wearing.^11,12^

Using a national survey of adults conducted from November 11 to December 9, 2020, this study examined individuals’ perceived chance of COVID-19 infection and severe consequences if infected, and how these varied among those with different numbers of risk factors; whether adults with more risk factors more often took actions associated with a lower risk of exposure, including avoiding crowds, social distancing, and wearing a mask in specific locations, such as grocery stores, and during activities, such as visiting with friends and hosting visitors at a residence; and whether individuals with more medical risk factors held opinions about mask wearing that were associated with more frequent mask wearing.

## Methods

### Data, Sample, and Survey Questions

The study used data from the Understanding America Study (UAS), which is a longitudinal internet-based survey representative of the civilian noninstitutionalized population 18 years and older in the US. The UAS selected households randomly from a list of household addresses in the US, and adults 18 years and older in these households were recruited through the mail. Ongoing surveys are collected over the internet, and sample members are provided a tablet and internet subscription if needed^13^; $20 was provided to respondents for each 30 minutes of interviewing. We used responses to questions administered in wave 18 of the UAS COVID-19 surveys from November 11 to December 9, 2020, which included 6084 respondents with a response rate of 77% for that wave.^14^ The American Association for Public Opinion Research (AAPOR) reporting guideline was used by UAS. The UAS was approved by the University of Southern California Institutional Review Board, and respondents provided informed consent online.

Using a validated visual linear scale,^15^ respondents reported their perceived belief about the percentage chance they will “get the coronavirus in the next three months,” and if they were to get the coronavirus, the percentage chance they will be “hospitalized (stay at least one night in the hospital) from it” and the percentage chance they would “die from it.” Table 1 provides the survey questions used to identify the following: 9 potentially higher infection risk activities undertaken in the last 7 days; mask wearing behavior while doing 6 of the 9 activities; whether a mask was worn anywhere in the past 7 days; and opinions about 12 aspects of mask wearing. Mask wearing while doing each of the 6 activities was asked only of those engaged in that activity, but whether an individual wore a mask anywhere in the past 7 days and opinions toward mask wearing were asked of all respondents.

**Table 1.** Survey Questions

Activity/mask wearing/opinion	Description
**Activities: In the last 7 days, have you done the following?**	
Bar/club	Gone out to a bar, club, or other place where people gather
Grocery/pharmacy	Gone to the grocery store or pharmacy
Visit friend’s home	Gone to a friend, neighbor, or relative’s residence (that is not your own)
Host visitors	Had visitors such as friends, neighbors, or relatives at your residence
Gathering of ≥10	Attended a gathering with 10 or more people, such as a reunion, wedding, funeral, birthday party, concert, or religious service
Left home for nonessential activity	Remained in your residence at all times, except for essential activities or exercise [reverse coded to make directionally consistent with other activities]
Share towel/utensil	Shared items like towels or utensils with other [we exclude from analyses those living alone]
<6 ft from coresident	Had close contact (within 6 ft) with people who live with you [we exclude from analyses those living alone]
<6 ft from noncoresident	Had close contact (within 6 ft) with people who do not live with you
**Mask wearing during specific activities: (ask for each “yes” response above) For each of the following activities, please indicate how often, if ever, you wore a mask or face covering. (Always/most of the time/sometimes/rarely/never/unsure)**	
Bar/club	When you went to a bar, club, or other place where people gather
Grocery/pharmacy	When you went to the grocery store or pharmacy
Visit friend’s home	When you went to a friend, neighbor, or other relative’s residence
Host visitors	When you had visitors such as friends, neighbors, or relatives at your residence
Gathering of ≥10	When you attended a gathering with 10 or more people
<6 ft from noncoresident	When you had close contact (within 6 ft) with people who do not live with you
**Mask wearing anywhere: Which of the following have you done in the last 7 days to keep yourself safe from coronavirus? Only consider actions that you took or decisions that you made personally.**	
Wore mask	Worn a mask or other face covering
**Opinions about masks: We would like to learn your general opinion about wearing a mask or face covering. Do you agree or disagree with each of the following statements? (Strongly disagree/disagree/neither agree nor disagree/agree/strongly agree)**	
Keeps me safe	Wearing a mask helps keep me safe from coronavirus
Keeps others safe	Wearing a mask helps keep others safe from coronavirus
Dangerous to my health	Wearing a mask is dangerous to my health
Political statement	Wearing a mask is a political statement
Not needed, I am not infected	Wearing a mask is not needed because I am not infected
Not needed if others healthy	Wearing a mask is not needed when I am with other people who are healthy
Others feel threatened	Others may feel threatened if I cover my face
Not needed if keep distance	I keep enough distance so that I do not need a mask
Cannot be forced to wear	We live in a free country and no one can force me to wear a mask
Cannot afford	I would like to wear a mask, but I cannot afford to buy one
Not needed, COVID-19 not serious	Wearing a mask is unnecessary because coronavirus is not a serious threat to people like me
Uncomfortable	A mask is too uncomfortable to wear

Nine medical conditions that the CDC has identified as associated with or possibly associated with severe illness from COVID-19 as of September 1, 2020,^1^ were measured in the UAS. Measurement was based on affirmative response to whether a physician, nurse, or health care professional has ever told the respondent they have chronic lung disease, kidney disease, heart disease, cancer, autoimmune disorder, diabetes, asthma, high blood pressure, or obesity. The CDC identifies those with multiple underlying conditions as having greater risk for severe illness if they become infected with COVID-19 and distinguishes among those with 0, 1, 2, and 3 or more conditions.^2^

The CDC identifies older age as a risk factor for severe illness from COVID-19.^1^ This analysis specified age as 18 to 59 years, 60 to 69 years, and 70 years and older after exploratory analyses showed relatively little variation in outcomes among those aged 18 to 59 years, distinct estimates for some outcomes for the latter 2 age groups, and insufficient sample size to disaggregate those 70 years and older. Race/ethnicity was specified as Hispanic, non-Hispanic White, non-Hispanic Black, and non-Hispanic other race. Education was 12 years or less, 13 to 15 years, and 16 or more years. Observations with missing data on any medical condition, age, sex, race/ethnicity, or education (n = 174) were excluded, resulting in 5910 people. Additional observations with missing data for outcome variables were infrequent (maximum of 1.2%) and were excluded only for analysis of the outcome for which it was missing. Sample size and descriptive statistics for all outcomes by risk factors are reported in eTables 1 and 2 in the Supplement.

### Statistical Analysis

Multivariable ordinary least squares regressions of perceived chance of infection, hospitalization if infected, and death if infected were estimated. For each of the 3 outcomes, 2 models were estimated: 1 including indicators for each of the 9 CDC medical risk factors and 1 instead including indicators for the number of medical risk factors (0, 1, 2, ≥3 conditions). All 6 models also included as explanatory variables age, sex, race/ethnicity, and education.

Multivariable logistic models were estimated for undertaking each activity, always wearing a mask at each activity, wore a mask anywhere, and whether strongly agree or agree with each of 12 opinions toward mask wearing. Multivariable Poisson regression was estimated for the number of activities undertaken over the past 7 days. Explanatory variables for each of these logistic and Poisson regressions included number of medical risk factors, age, sex, race/ethnicity, and education. Additional multivariable logistic models of mask wearing also were estimated with these explanatory variables as well as indicator variables for agreeing or strongly agreeing with each of the 12 mask-wearing opinions.

For each of these models, the adjusted outcome for each risk factor (specific medical condition or number of medical conditions and age group) was calculated by averaging the predicted value from the regression model for each observation holding all explanatory variables at their observed values except for the individual risk factor. Tests for differences in outcomes between those with vs without the risk factor were based on tests of significance of the risk factor in the corresponding multivariable regression. Unadjusted outcomes (eTables 1 and 2 in the Supplement) infer the same primary conclusions as adjusted outcomes. We interpret differences as being statistically significant if *P* < .05 (2-tailed tests).

Responses for the 3 perceived-chance variables were not normally distributed; therefore, supplemental analyses examined sensitivity of the conclusions to the use of unconditional median regression^16^ instead of ordinary least squares regression of perceived chances. The UAS final poststratification sample weight^17^ and Stata, version 16 (StataCorp LLC) software were used for all analyses.

## Results

The response rate of UAS wave 18 was 77%, with 6084 respondents. The weighted demographic characteristics were similar to those based on the US National Health Interview Survey,^18^ with 48.4% being men and 69.4% being aged 18 to 59 years (eTable 3 in the Supplement). Disease prevalence ranged from 2.6% for kidney disease to 30.9% for high blood pressure (eTable 3 in the Supplement). None of the 9 medical risk factors were reported for 47.4%, and 27.4%, 14.2%, and 11.0% had 1, 2, and 3 or more medical risk factors, respectively. A positive test result for SARS-CoV-2 since last interview (approximately 2-4 weeks) was reported for 58 participants (0.98%).

All but 2 of the 9 medical risk factors were associated with elevated perceived chance of hospitalization or death (high blood pressure and kidney disease; Table 2). Adjusted mean perceived percentage chance of hospitalization if infected ranged from 23.9% (95% CI, 22.2%-25.5%) for high blood pressure to 40.4% (95% CI, 34.6%-46.2%) for chronic lung disease. Adjusted mean perceived chance of infection was elevated significantly for those with diabetes (27.4%; 95% CI, 24.8%-29.9%; *P* = .01), autoimmune disorder (28.7%; 95% CI, 25.6-31.8; *P* = .005), heart disease (27.4%; 95% CI, 24.3%-30.5%; *P* = .04), and chronic lung disease (31.0%; 95% CI, 26.9%-35.1%; *P* < .001). Adults aged 70 years and older (relative to those aged 18-59 years) had elevated adjusted mean perceived chance of hospitalization (31.4%; 95% CI, 28.5%-34.3%; *P* < .001) and death (25.6%; 95% CI, 22.8%-28.4%; *P* < .001) if infected but reduced perceived risk of becoming infected (21.7%; 95% CI, 19.7%-23.7%; *P* = .003). For all 3 perceived risks, the association with number of conditions was statistically significant based on both ordinary least squares and unconditional median regression (eTables 4-6 in the Supplement).

**Table 2.** Adjusted Mean Perceived Percentage Chance of Infection With COVID-19 in the Next 3 Months and Hospitalization and Death If Infected, by CDC Risk Factor

Characteristic	Perceived chance, % (95% CI)^a^					
	Infection	*P* value	Hospitalized, if infected	*P* value	Dying, if infected	*P* value
**CDC risk factor**^b^^,^^c^						
No. of conditions						
0	22.7 (21.5-24.0)	NA	17.6 (16.4-18.8)	NA	13.6 (12.5-14.7)	NA
1	23.7 (22.3-25.2)	.31	22.4 (20.7-24.1)	<.001	17.7 (16.2-19.3)	<.001
2	25.7 (23.7-27.8)	.02	25.9 (23.6-28.3)	<.001	19.2 (17.0-21.4)	<.001
≥3	31.0 (28.6-33.4)	<.001	41.8 (38.7-45.0)	<.001	34.3 (31.2-37.5)	<.001
Age, y						
18-59	25.2 (24.1-26.2)	NA	20.4 (19.4-21.5)	NA	15.6 (14.6-16.6)	NA
60-69	23.1 (21.4-24.7)	.04	25.6 (23.5-27.6)	<.001	20.8 (18.8-22.8)	<.001
≥70	21.7 (19.7-23.7)	.003	31.4 (28.5-34.3)	<.001	25.6 (22.8-28.4)	<.001
**Specific condition**^d^^,^^e^						
High blood pressure	23.9 (22.4-25.4)	.50	23.9 (22.2-25.5)	.13	18.2 (16.6-19.8)	.59
Obesity	25.8 (23.9-27.7)	.10	26.5 (24.3-28.6)	<.001	19.6 (17.6-21.6)	.05
Asthma	25.0 (22.7-27.3)	.53	26.8 (24.2-29.5)	.001	20.5 (18.1-22.9)	.02
Kidney disease	28.7 (23.6-33.8)	.09	28.1 (22.0-34.1)	.08	22.5 (16.1-28.8)	.15
Cancer	27.3 (24.0-30.5)	.07	29.4 (25.6-33.2)	<.001	24.2 (20.4-28.1)	.001
Diabetes	27.4 (24.8-29.9)	.01	30.2 (27.1-33.2)	<.001	23.7 (20.8-26.5)	<.001
Autoimmune disorder	28.7 (25.6-31.8)	.005	30.3 (26.2-34.4)	<.001	24.3 (20.6-28.0)	<.001
Heart disease	27.4 (24.3-30.5)	.041	31.8 (27.5-36.1)	<.001	28.3 (23.9-32.7)	<.001
Chronic lung disease	31.0 (26.9-35.1)	<.001	40.4 (34.6-46.2)	<.001	37.3 (31.3-43.2)	<.001

Abbreviations: CDC, Centers for Disease Control and Prevention; NA, not applicable.

^a^ Adjusted perceived percentage based on multivariable ordinary least squares regressions reported in eTables 4-6 in the Supplement, which control for age, sex, race/ethnicity, and education.

^b^ *P* values are associated with the coefficients on number of conditions relative to 0 conditions and age relative to those aged 18 to 59 y.

^c^ Estimates are based on models that also include indicators for number of conditions.

^d^ *P* values are associated with the coefficients relative to having vs not having the given condition.

^e^ Estimates are based on analogous models but include indicators of specific conditions instead of number of conditions.

Having 3 or more relative to 0 medical risk factors was associated with 9.4% fewer activities in the past 7 days (adjusted rate of 2.83; 95% CI, 2.66-2.99; vs 3.12; 95% CI, 3.02-3.22; *P* = .005) and a lower likelihood of visiting a friend’s home (adjusted proportion of 0.338; 95% CI, 0.289-0.387; vs 0.40; 95% CI, 0.376-0.428; *P* = .03) or leaving home for nonessential activities (adjusted proportion of 0.447; 95% CI, 0.397-0.497; vs 0.607; 95% CI, 0.591-0.623; *P* < .001; Table 3). Relative to adults aged 18 to 59 years, adults 70 years and older engaged in 17.8% fewer activities in the past 7 days in total (adjusted mean, 2.65; 95% CI, 2.51-2.79; vs 3.23; 95% CI, 3.15-3.31; *P* < .001) and were less likely to undertake 5 of the 9 specific activities (bar/club; visit friend’s home; host visitors; left home, nonessential; within 6 ft of noncoresident).

**Table 3.** Adjusted Proportion Completing Various Activities and Number of Activities in the Past 7 Days, by CDC Risk Factor

Activity	Proportion (95% CI)^a^						
	No. of conditions				Age, y		
	0	1	2	≥3	18-59	60-69	≥70
Visit bar/club	0.096 (0.081-0.111)	0.109 (0.088-0.131)	0.073 (0.051-0.095)	0.089 (0.058-0.119)	0.106 (0.093-0.120)	0.089 (0.067-0.111)	0.054 (0.035-0.073)
*P* value^b^	NA	.31	.12	.69	NA	.21	<.001
Visit grocery store/pharmacy	0.808 (0.787-0.830)	0.839 (0.814-0.864)	0.832 (0.797-0.866)	0.789 (0.747-0.832)	0.806 (0.788-0.824)	0.870 (0.844-0.896)	0.811 (0.773-0.849)
*P* value^b^	NA	.08	.28	.44	NA	<.001	.82
Visit friend’s home	0.402 (0.376-0.428)	0.420 (0.387-0.453)	0.385 (0.341-0.430)	0.338 (0.289-0.387)	0.418 (0.396-0.440)	0.378 (0.340-0.416)	0.316 (0.274-0.358)
*P* value^b^	NA	.41	.53	.03	NA	.08	<.001
Host visitors	0.418 (0.392-0.444)	0.429 (0.397-0.462)	0.426 (0.381-0.471)	0.430 (0.380-0.480)	0.438 (0.416-0.460)	0.397 (0.360-0.434)	0.386 (0.343-0.430)
*P* value^b^	NA	.60	.76	.68	NA	.07	.04
Gathering of ≥10 people	0.155 (0.135-0.174)	0.153 (0.129-0.177)	0.141 (0.108-0.174)	0.137 (0.100-0.174)	0.156 (0.139-0.172)	0.137 (0.110-0.165)	0.140 (0.108-0.172)
*P* value^b^	NA	.92	.51	.42	NA	.29	.42
Left home, nonessential	0.607 (0.581-0.633)	0.573 (0.541-0.606)	0.534 (0.490-0.579)	0.447 (0.397-0.497)	0.627 (0.605-0.648)	0.481 (0.443-0.519)	0.388 (0.345-0.431)
*P* value^b^	NA	.11	.006	<.001	NA	<.001	<.001
Share towel/utensil^c^	0.299 (0.272-0.325)	0.298 (0.265-0.332)	0.282 (0.236-0.328)	0.258 (0.206-0.311)	0.303 (0.281-0.325)	0.243 (0.206-0.280)	0.297 (0.248-0.347)
*P* value^b^	NA	.98	.56	.20	NA	.01	.85
<6 ft of coresident^c^	0.873 (0.853-0.893)	0.914 (0.893-0.936)	0.905 (0.874-0.936)	0.875 (0.836-0.915)	0.883 (0.868-0.899)	0.901 (0.873-0.929)	0.913 (0.878-0.949)
*P* value^b^	NA	.01	.11	.93	NA	.29	.17
<6 ft of noncoresident	0.628 (0.602-0.654)	0.668 (0.637-0.698)	0.638 (0.595-0.681)	0.606 (0.557-0.655)	0.670 (0.649-0.690)	0.580 (0.542-0.618)	0.541 (0.495-0.587)
*P* value^b^	NA	.05	.68	.45	NA	<.001	<.001
No. of activities, mean (95% CI)^d^	3.12 (3.02-3.22)	3.20 (3.08-3.32)	3.03 (2.87-3.18)	2.83 (2.66-2.99)	3.23 (3.15-3.31)	2.94 (2.81-3.07)	2.65 (2.51-2.79)
*P* value^b^	NA	.28	.32	.005	NA	<.001	<.001

Abbreviations: CDC, Centers for Disease Control and Prevention; NA, not applicable.

^a^ Adjusted proportion and adjusted mean No. of activities based on multivariable models controlling for number of conditions, age, sex, race/ethnicity, and education reported in eTable 7 in the Supplement.

^b^ *P* values are associated with the coefficients on number of conditions relative to 0 conditions and age relative to 18-59 y in these multivariable models.

^c^ Among those who live with someone else.

^d^ Excludes shared towel/utensils and <6 ft from coresident because some people live alone.

For only 1 activity (visiting a grocery store or pharmacy) were participants with 3 or more medical risk factors more likely to always wear a mask than those with no conditions; the difference between groups for attending a gathering of 10 or more was sizable but not statistically significant (adjusted proportion of 0.350; 95% CI, 0.228-0.473; vs 0.228; 95% CI, 0.173-0.283; *P* = .06; Table 4). For 5 of the 6 activities, no more than 38.4% always wore a mask while doing the activity even among adults with 3 or more medical risk factors. Mask wearing was most common at the grocery store or pharmacy (86.9% [95% CI, 83.0%-90.7%] among adults with ≥3 medical risk factors). For 3 of 6 activities (bar/club, grocery store/pharmacy, gathering of ≥10 people), adults 70 years and older were more likely to always wear a mask than adults aged 18 to 59 years. But with the exception of grocery store/pharmacy, no more than 45.7% of adults 70 years and older always wore a mask during any of the activities. Despite low rates of mask wearing for most specific activities, nearly all adults wore a mask at some point in the past 7 days (88.0%-94.5%, depending on the risk factor; Table 4).

**Table 4.** Adjusted Proportion Wearing a Mask in the Past 7 Days, by CDC Risk Factor

Mask wearing	Proportion (95% CI)^a^						
	No. of conditions				Age, y		
	0	1	2	≥3	18-59	60-69	≥70
Wore a mask somewhere in past 7 d	0.880 (0.862-0.897)	0.920 (0.901-0.939)	0.922 (0.897-0.947)	0.933 (0.906-0.961)	0.883 (0.868-0.898)	0.944 (0.926-0.962)	0.945 (0.923-0.967)
*P* value^b^	NA	.004	.02	.008	NA	<.001	<.001
Always wore a mask in past 7 d while at							
Bar/club	0.219 (0.150-0.287)	0.305 (0.210-0.400)	0.384 (0.235-0.532)	0.172 (0.068-0.276)	0.210 (0.155-0.265)	0.390 (0.262-0.519)	0.437 (0.271-0.603)
*P* value^b^	NA	.14	.03	.49	NA	.008	.005
Grocery store/pharmacy	0.814 (0.791-0.838)	0.823 (0.795-0.851)	0.858 (0.823-0.893)	0.869 (0.830-0.907)	0.802 (0.782-0.822)	0.881 (0.853-0.909)	0.887 (0.856-0.919)
*P* value^b^	NA	.64	.06	.03	NA	<.001	<.001
Visiting friend’s home	0.076 (0.054-0.098)	0.094 (0.063-0.124)	0.126 (0.078-0.173)	0.112 (0.056-0.168)	0.085 (0.066-0.104)	0.105 (0.067-0.142)	0.116 (0.063-0.170)
*P* value^b^	NA	.34	.04	.19	NA	.33	.24
Hosting visitors	0.063 (0.042-0.084)	0.065 (0.040-0.091)	0.114 (0.070-0.158)	0.091 (0.049-0.133)	0.070 (0.052-0.088)	0.084 (0.053-0.115)	0.089 (0.050-0.129)
*P* value^b^	NA	.89	.02	.21	NA	.43	.36
Gathering of ≥10 people	0.228 (0.173-0.283)	0.308 (0.233-0.382)	0.372 (0.256-0.488)	0.350 (0.228-0.473)	0.216 (0.169-0.262)	0.445 (0.350-0.540)	0.457 (0.347-0.567)
*P* value^b^	NA	.08	.02	.06	NA	<.001	<.001
<6 ft of noncoresident	0.269 (0.240-0.299)	0.271 (0.235-0.307)	0.350 (0.295-0.404)	0.280 (0.220-0.340)	0.274 (0.250-0.298)	0.302 (0.256-0.347)	0.304 (0.249-0.359)
*P* value^b^	NA	.95	.009	.76	NA	.30	.33

^a^ Adjusted proportions based on multivariable logistic models controlling for number of conditions, age, sex, race/ethnicity, and education reported in eTable 8 in the Supplement.

^b^ *P* values are associated with the coefficients on number of conditions relative to 0 conditions and age relative to 18-59 y in these multivariable models.

Odds ratios from multivariable logistic regression indicate that relative to adults with no medical risk factors, adults with 3 or more factors were more likely to hold opinions that should be associated with higher mask wearing (keeps me safe, keeps others safe; Figure) and were less likely to hold some opinions that should be associated with lower mask wearing (not needed if others are healthy, not needed if not infected, not needed if keep distance, COVID-19 not serious). Adults with 3 or more medical risk factors did not differ from those with no risk factors on other opinions that should be associated with lower mask wearing (is dangerous, is threatening to others, cannot force me to wear, cannot afford, is uncomfortable). Those with 3 or more medical risk factors also were less likely to believe that mask wearing is a political statement (Figure). Compared with adults aged 18 to 59 years, those 70 years and older were more likely to hold favorable opinions about mask wearing and less likely to hold unfavorable opinions in all but 1 instance (cannot afford). When the 12 opinions toward mask wearing were added as explanatory variables to the models of mask wearing, the difference between 3 or more conditions vs 0 conditions or aged 70 years and older vs aged 18 to 59 years did not widen for any of the mask-wearing outcomes (eTable 10 in the Supplement).

**Figure.**
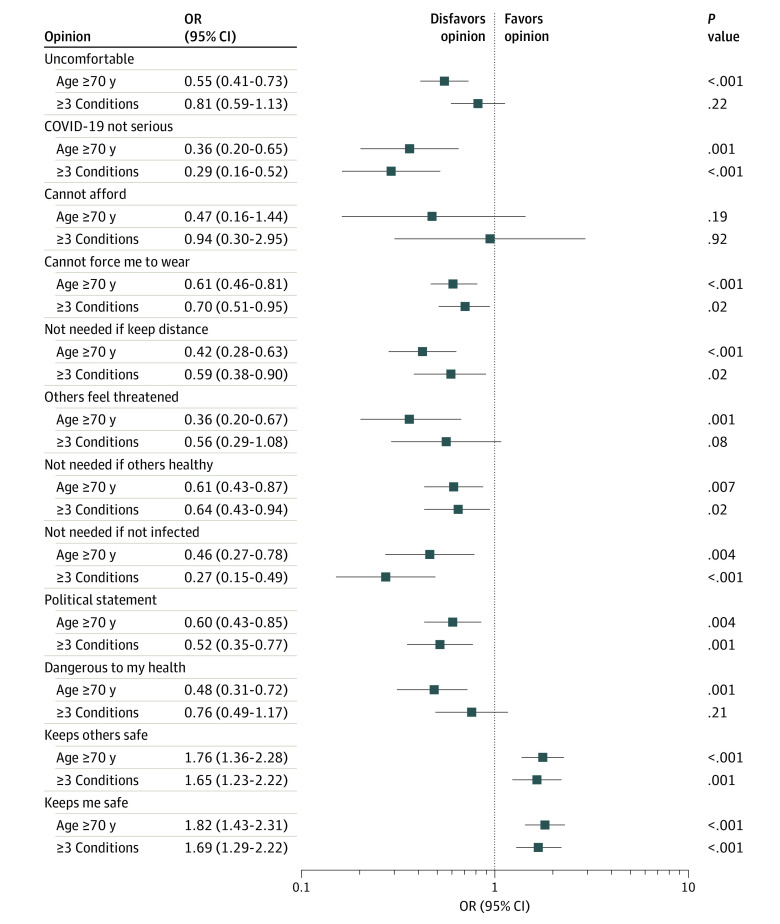
Participants’ Opinions Regarding Masks Associated With Participant Risk Factors and Age Multivariable logistic regressions are reported in eTable 9 in the Supplement and include explanatory variables for number of conditions, age, sex, race/ethnicity, and education. OR indicates odds ratio.

## Discussion

Using a nationally representative survey of US adults, we found that older adults and those with medical risk factors associated with severe COVID-19 complications understood and reported their higher risk. We examined 9 medical conditions the CDC identified as being associated with severe complications from COVID-19.^1^ For 7 of 9 conditions, the mean perceived chance of hospitalization and of death if infected was higher among adults with the medical condition than those without the condition; exceptions were high blood pressure and kidney disease. Adults with more medical conditions perceived a higher chance of infection, but only some specific conditions were associated with higher perceived risk. Older adults did not perceive a higher chance of infection.

Having a greater number of medical conditions was associated with engaging in fewer activities per week where adults might be exposed to the virus, but the difference was modest in size (9.4% fewer activities among adults with ≥3 vs 0 conditions), and there was no association for some high-exposure activities, including being within 6 feet of someone the person did not live with. Adults 70 years and older engaged in 17.8% fewer activities than adults aged 18 to 59 years. Engaging in fewer activities may have been 1 factor influencing differences in perceived risk of infection across groups. These conclusions are generally consistent with findings from earlier in the pandemic^8^ that adults who believe they are at high risk of complications from the virus take more safety precautions.

Wearing a mask sometime in the last 7 days was nearly universal, but persistent use was uncommon for most specific activities, including activities that were done by many adults each week. For only 1 of 6 activities examined (shopping at a grocery store or pharmacy) did more than half always wear a mask during the activity. The high prevalence of mask wearing at grocery stores and pharmacies may reflect mask requirements at these businesses. Two-fifths of adults visited a friend’s home and 42% hosted visitors at their homes in the past 7 days, but even among adults with 3 or more medical risk factors, only 11% and 9% always wore a mask when doing these activities, respectively. More generally, nearly two-thirds of adults said they had been within 6 feet of someone who they did not live with, and when they were, less than one-third always wore a mask. In most settings, adults with 3 or more medical risk factors were not more likely to always wear a mask. Moreover, the lack of consistently greater mask use among adults who were more susceptible was not because they were more likely to believe wearing a mask is uncomfortable, costly, dangerous, ineffective, or threatening to others; adults with risk factors tended to have opinions about masks that should elevate their mask wearing.

Visiting with friends or family or being in close contact with individuals who do not live in the household without wearing a mask increases exposure to the virus, but the importance for the overall spread of the virus remains unknown. Some evidence comes from Maryland, where contact tracing from July indicates that 44% of infected patients had attended a family gathering.^19^ In settings such as family gatherings, rising infection rates among children^20^ and young adults^21^ may have intergenerational consequences. Family gatherings may be difficult settings in which to wear masks and ask that others do so as well. The low prevalence of mask wearing in small gatherings may indicate the role of social networks and peers in influencing behavior.^22^ In addition, mask wearing with family and friends may be difficult because of the importance of facial expressions for communication among those who know each other well. In one study,^23^ most participants stated that people most important to them want them to wear a face covering; however, individuals may not equate being with family and friends as being in public. A better understanding is needed of why so many people, even those at higher risk for complications, do not wear masks consistently when in these settings. Such evidence is necessary to inform the development of strategies to improve mask wearing during these common activities.

### Limitations

The study has potential limitations. Prepandemic differences in health knowledge and behaviors between adults with various health conditions were not examined, and they and other factors not measured in the UAS may be associated with the outcomes and exposures. Risk associated with some specific activities may have been reduced by ways not measured in the survey, such as physical distancing or visiting with friends outside, eating at restaurants outdoors, or knowing that individuals with whom the person is interacting had recently tested negative. Although we use the number of specific activities as a summary measure, all activities do not carry the same level of transmission risk.^24,25^ Not all medical risk factors were measured or measured with as much specificity as identified in CDC’s list of risk factors, and the high-risk institutionalized population was not studied. Measuring obesity as having been told by a health care professional instead of calculating obesity based on height and weight led to an underestimate of obesity prevalence (eTable 3 in the Supplement). If health care professionals are more likely to discuss obesity with more severely obese adults, the differences we report in perceived risks associated with obesity might be overstated. Reporting in this study of activities and mask wearing in the past 7 days may have been subject to recall bias. COVID-19–related knowledge and behaviors may have changed during the pandemic. Finally, prior research has found that subjective assessments of the likelihood of negative events can be overstated,^26^ which was likely the case here, although evidence that subjective expectations about life expectancy are associated with mortality suggests that individuals’ perceptions have some validity.^4^

## Conclusions

This study documented how perception of risk of infection and severe complications from COVID-19 were associated with underlying reported health. That adults with most CDC-identified medical conditions and of older age perceived elevated risks of complications suggests that at least some parts of public health messaging regarding COVID-19 have been successfully communicated. We found some evidence that adults with conditions that increase risk of severe complications from COVID-19 were more likely to practice protective behaviors. But the low rates of mask wearing during activities that were undertaken frequently, even among those most susceptible based on medical conditions and age, point to the need to better identify how individuals understand the risks and potential benefits of this nonpharmacologic intervention and to translate this knowledge into effective efforts to increase consistent mask wearing in all settings where risk of exposure may be elevated.

## Notes

Supplement. **eTable 1. **Descriptive statistics of outcomes, by number of conditions **eTable 2. **Descriptive statistics of outcomes, by age group **eTable 3. **Comparison of sample characteristics based on Understanding America Study (UAS) and National Health Interview Survey (NHIS) **eTable 4. **OLS and unconditional median regressions of perceived percent chance of infection in next 3 months **eTable 5. **OLS and unconditional median regressions of perceived percent chance of hospitalization if infected **eTable 6. **OLS and unconditional median regressions of perceived percent chance of death if infected **eTable 7. **Models of activities undertaken in the past 7 days **eTable 8. **Logistic models of mask wearing in the past 7 days **eTable 9. **Logistic models of whether agree or strongly agree about various aspects of wearing a mask **eTable 10. **Adjusted proportion wearing a mask in the past 7 days, by CDC risk factor [95% CI], with controls for opinions about masks
