# Supplementary material for: Association Between Risk Factors for Complications From COVID-19, Perceived Chances of Infection and Complications, and Protective Behavior in the US
Source: JAMA Netw Open. 2021 Mar 31;4(3):e213984. doi: 10.1001/jamanetworkopen.2021.3984 (PMC8013830; doi:10.1001/jamanetworkopen.2021.3984)
Supplement: Supplement. — eTable 1. Descriptive statistics of outcomes, by number of conditions eTable 2. Descriptive statistics of outcomes, by age group eTable 3. Comparison of sample characteristics based on Understanding America Study (UAS) and National Health Interview Survey (NHIS) eTable 4. OLS and unconditional median regressions of perceived percent chance of infection in next 3 months eTable 5. OLS and unconditional median regressions of perceived percent chance of hospitalization if infected eTable 6. OLS and unconditional median regressions of perceived percent chance of death if infected eTable 7. Models of activities undertaken in the past 7 days eTable 8. Logistic models of mask wearing in the past 7 days eTable 9. Logistic models of whether agree or strongly agree about various aspects of wearing a mask eTable 10. Adjusted proportion wearing a mask in the past 7 days, by CDC risk factor [95% CI], with controls for opinions about masks [file jamanetwopen-e213984-s001.pdf]

# Supplemental Online Content

Schoeni RF, Wiemers EE, Seltzer JA, Langa KM. Association between risk factors for complications from COVID-19, perceived chances of infection and complications, and protective behavior in the US. *JAMA Netw Open*. 2021;4(3):e213984.
doi:10.1001/jamanetworkopen.2021.3984

**eTable 1.** Descriptive statistics of outcomes, by number of conditions

**eTable 2.** Descriptive statistics of outcomes, by age group

**eTable 3.** Comparison of sample characteristics based on Understanding America Study (UAS) and National Health Interview Survey (NHIS)

**eTable 4.** OLS and unconditional median regressions of perceived percent chance of infection in next 3 months

**eTable 5.** OLS and unconditional median regressions of perceived percent chance of hospitalization if infected

**eTable 6.** OLS and unconditional median regressions of perceived percent chance of death if infected

**eTable 7.** Models of activities undertaken in the past 7 days

**eTable 8.** Logistic models of mask wearing in the past 7 days

**eTable 9.** Logistic models of whether agree or strongly agree about various aspects of wearing a mask

**eTable 10.** Adjusted proportion wearing a mask in the past 7 days, by CDC risk factor [95% CI], with controls for opinions about masks

This supplemental material has been provided by the authors to give readers additional information about their work.

© 2021 Schoeni RF et al. *JAMA Network Open.*

eTable 1. Descriptive statistics of outcomes, by number of conditions

| | All adults | | 0 conditions | | 1 condition | | 2 conditions | | >=3 conditions | | 0 vs >=3 |
|---|---|---|---|---|---|---|---|---|---|---|---|
| | n | %/mean(StdDev) | n | %/mean(StdDev) | n | %/mean(StdDev) | n | %/mean(StdDev) | n | %/mean(StdDev) | p-value |
| Chance get infected | 5900 | 24.3 (22.8) | 2648 | 23.3 (22.4) | 1643 | 23.4 (22.3) | 896 | 25.0 (22.5) | 713 | 30.2 (24.0) | <0.001 |
| Chance hospitalized | 5899 | 22.8 (26.1) | 2646 | 16.3 (20.5) | 1643 | 22.7 (25.7) | 896 | 27.6 (28.1) | 714 | 44.7 (33.2) | <0.001 |
| Chance die | 5900 | 17.8 (24.7) | 2647 | 12.4 (19.7) | 1643 | 18. 0 (24.3) | 896 | 20.7 (26.0) | 714 | 37.2 (33.5) | <0.001 |
| <u>Activities last 7 days</u> | | | | | | | | | | | |
| Bar/club | 5862 | 9.6% | 2631 | 10.0% | 1631 | 10.8% | 888 | 7.0% | 712 | 8.0% | 0.23 |
| Grocery/pharmacy | 5859 | 81.8% | 2625 | 80.4% | 1630 | 84.9% | 891 | 83.8% | 713 | 79.7% | 0.76 |
| Visit friend | 5858 | 39.8% | 2628 | 41.2% | 1628 | 41.4% | 892 | 37.6% | 710 | 32.2% | 0.001 |
| Had visitors | 5869 | 42.4% | 2631 | 42.0% | 1633 | 42.7% | 893 | 42.7% | 712 | 42.9% | 0.75 |
| Gathering of 10+ | 5859 | 15.0% | 2627 | 15.5% | 1630 | 15.3% | 890 | 14.1% | 712 | 13.5% | 0.34 |
| Left home, nonessential | 5841 | 56.9% | 2617 | 63.0% | 1622 | 56.5% | 891 | 50.9% | 711 | 40.2% | <0.001 |
| Share towel/utensil[a] | 4632 | 29.2% | 2113 | 30.8% | 1300 | 29.3% | 688 | 27.6% | 531 | 23.9% | 0.02 |
| <6ft of coresident[a] | 4652 | 88.9% | 2124 | 86.8% | 1308 | 91.4% | 690 | 91.3% | 530 | 88.1% | 0.55 |
| <6ft from noncoresident | 5841 | 63.8% | 2616 | 64.2% | 1629 | 66.2% | 889 | 62.5% | 707 | 57.7% | 0.02 |
| Number of activities[b] | 5744 | 3.10 (1.78) | 2572 | 3.17 (1.76) | 1599 | 3.18 (1.80) | 873 | 2.98 (1.72) | 700 | 2.74 (1.71) | <0.001 |
| <u>Always wore mask when…</u> | | | | | | | | | | | |
| Bar/club | 522 | 25.9% | 259 | 20.0% | 144 | 31.2% | 64 | 42.6% | 55 | 21.2% | 0.86 |
| Grocery/pharmacy | 4857 | 82.9% | 2165 | 80.7% | 1384 | 82.8% | 749 | 86.4% | 559 | 87.4% | 0.002 |
| Visit friend | 2287 | 9.1% | 1106 | 7.3% | 638 | 9.6% | 331 | 13.1% | 212 | 11.8% | 0.13 |
| Had visitors | 2414 | 7.5% | 1093 | 5.9% | 670 | 6.8% | 363 | 12.2% | 288 | 9.7% | 0.14 |
| Gathering of 10+ | 801 | 28.5% | 390 | 19.8% | 229 | 31.7% | 102 | 43.1% | 80 | 42.3% | 0.004 |
| <6ft of someone | 3704 | 28.2% | 1711 | 26.7% | 1044 | 27.5% | 545 | 35.0% | 404 | 27.8% | 0.74 |
| <u>Action taken to keep safe in past 7 days</u> | | | | | | | | | | | |
| Wore mask | 5901 | 90.1% | 2649 | 87.3% | 1643 | 92.2% | 895 | 92.7% | 714 | 93.9% | <0.001 |
| <u>Agree or strongly agree about masks</u> | | | | | | | | | | | |
| Make me safe | 5897 | 67.1% | 2649 | 63.1% | 1643 | 69.3% | 892 | 69.4% | 713 | 75.6% | <0.001 |
| Make others safe | 5903 | 72.7% | 2651 | 68.7% | 1642 | 75.2% | 896 | 76.2% | 714 | 79.4% | <0.001 |
| Dangerous to wear | 5902 | 9.7% | 2651 | 11.5% | 1642 | 7.5% | 895 | 9.2% | 714 | 8.1% | 0.04 |
| Political statement | 5902 | 11.7% | 2650 | 14.0% | 1642 | 10.1% | 896 | 10.2% | 714 | 7.6% | <0.001 |
| Not need if not infected | 5900 | 7.1% | 2650 | 9.6% | 1642 | 5.3% | 895 | 5.6% | 713 | 2.5% | <0.001 |
| Not need if other healthy | 5905 | 12.2% | 2652 | 15.5% | 1644 | 8.9% | 895 | 10.0% | 714 | 9.6% | 0.001 |
| Makes other threatened | 5904 | 4.8% | 2650 | 5.2% | 1644 | 5.0% | 896 | 4.8% | 714 | 2.4% | 0.003 |
| Not need if keep distance | 5903 | 10.5% | 2650 | 12.7% | 1643 | 9.7% | 896 | 8.1% | 714 | 6.2% | <0.001 |
| Can't make me wear | 5901 | 19.3% | 2648 | 21.4% | 1644 | 17.5% | 895 | 18.6% | 714 | 15.6% | 0.005 |
| Can't afford | 5902 | 1.7% | 2649 | 1.9% | 1643 | 1.8% | 896 | 1.5% | 714 | 1.2% | 0.37 |
| C-19 not serious | 5903 | 5.7% | 2651 | 8.4% | 1644 | 3.5% | 895 | 3.5% | 713 | 2.2% | <0.001 |
| Uncomfortable | 5903 | 19.0% | 2649 | 21.3% | 1644 | 18.1% | 896 | 15.4% | 714 | 16.2% | 0.02 |

[a] Among those who live with someone else. [b] Excludes shared towel / utensils and <6ft from coresident because some people live alone.

© 2021 Schoeni RF et al. *JAMA Network Open.*

eTable 2. Descriptive statistics of outcomes, by age group

| | 18-59 | | 60-69 | | >=70 | | 18-59 vs >=70 |
|---|---|---|---|---|---|---|---|
| | N | %/mean(StdDev) | N | %/mean(StdDev) | N | %/mean(StdDev) | p-value |
| Chance get infected | 4074 | 24.8 (22.5) | 1233 | 23.9 (22.9) | 865 | 22.7 (23.0) | 0.06 |
| Chance hospitalized | 4072 | 19.1 (22.4) | 1234 | 28.1 (30.1) | 865 | 35.4 (35.3) | <0.001 |
| Chance die | 4071 | 14.6 (20.9) | 1233 | 22.8 (29.3) | 865 | 28.5 (34.1) | <0.001 |
| Activities last 7 days | | | | | | | |
| Bar/club | 4040 | 10.3% | 1233 | 9.4% | 864 | 6.1% | 0.001 |
| Grocery/pharmacy | 4044 | 80.4% | 1234 | 87.2% | 866 | 81.8% | 0.48 |
| Visit friend | 4039 | 41.5% | 1230 | 38.3% | 865 | 32.2% | <0.001 |
| Had visitors | 4042 | 42.7% | 1233 | 41.5% | 863 | 41.6% | 0.64 |
| Gathering of 10+ | 4035 | 15.4% | 1232 | 14.0% | 865 | 14.6% | 0.66 |
| Left home, nonessential | 4023 | 62.6% | 1229 | 48.2% | 860 | 39.0% | <0.001 |
| Share towel/utensil[a] | 3381 | 30.2% | 907 | 24.4% | 617 | 29.8% | 0.87 |
| <6ft of coresident[a] | 3397 | 87.5% | 913 | 91.5% | 620 | 93.4% | <0.001 |
| <6ft from noncoresident | 4022 | 66.1% | 1224 | 59.5% | 858 | 57.3% | <0.001 |
| Number of activities[b] | 3952 | 3.20 (.1.74) | 1215 | 2.98 (1.80) | 846 | 2.73 (1.77) | <0.001 |
| Always wore mask when… | | | | | | | |
| Bar/club | 398 | 21.5% | 102 | 38.1% | 48 | 39.0% | 0.05 |
| Grocery/pharmacy | 3363 | 80.4% | 1038 | 87.6% | 710 | 88.8% | <0.001 |
| Visit friend | 1939 | 8.7% | 525 | 10.2% | 310 | 10.2% | 0.58 |
| Had visitors | 1818 | 7.2% | 561 | 7.7% | 407 | 8.5% | 0.55 |
| Gathering of 10+ | 730 | 21.7% | 180 | 44.6% | 112 | 45.6% | <0.001 |
| <6ft of someone | 2681 | 28.0% | 742 | 28.8% | 457 | 28.5% | 0.86 |
| Action taken to keep safe | | | | | | | |
| Wore mask | 4075 | 88.2% | 1233 | 94.3% | 866 | 94.8% | <0.001 |
| Agree or strongly agree about masks | | | | | | | |
| Make me safe | 4076 | 63.7% | 1233 | 74.9% | 865 | 74.5% | <0.001 |
| Make others safe | 4073 | 69.4% | 1234 | 80.7% | 865 | 79.7% | <0.001 |
| Dangerous to wear | 4075 | 10.9% | 1234 | 7.7% | 865 | 5.8% | <0.001 |
| Political statement | 4077 | 12.6% | 1233 | 10.6% | 866 | 8.4% | 0.002 |
| Not need if not infected | 4076 | 8.3% | 1233 | 4.5% | 865 | 3.8% | <0.001 |
| Not need if others healthy | 4074 | 13.5% | 1234 | 10.0% | 866 | 8.8% | 0.001 |
| Makes others threatened | 4074 | 5.7% | 1234 | 3.2% | 866 | 2.1% | <0.001 |
| Not need if keep distance | 4075 | 12.5% | 1234 | 6.6% | 866 | 5.6% | <0.001 |
| Can't make me wear | 4075 | 20.5% | 1234 | 17.5% | 866 | 15.5% | 0.006 |
| Can't afford | 4077 | 2.3% | 1234 | 0.1% | 866 | 1.0% | 0.02 |
| C-19 not serious | 4073 | 7.0% | 1234 | 3.0% | 866 | 2.4% | <0.001 |
| Uncomfortable | 4075 | 21.3% | 1233 | 14.6% | 865 | 13.1% | <0.001 |

[a] Among those who live with someone else. [b] Excludes shared towel / utensils and <6ft from coresident because some people live alone.

© 2021 Schoeni RF et al. *JAMA Network Open.*

## eTable 3. Comparison of sample characteristics based on Understanding America Study (UAS) and National Health Interview Survey (NHIS)

| | UAS, November 11-December 9, 2020 (n=5910) | NHIS, 2018 (n=53684) | p-value for difference between surveys |
|---|---|---|---|
| | Proportion | Proportion | |
| Diabetes | 0.125 | 0.123 | 0.78 |
| Cancer* | 0.061 | 0.094 | <0.001 |
| Heart disease | 0.062 | 0.081 | <0.001 |
| High blood pressure | 0.309 | 0.309 | 0.97 |
| Asthma | 0.116 | 0.131 | 0.02 |
| Chronic lung disease | 0.042 | 0.046 | 0.38 |
| Kidney disease* | 0.026 | 0.023 | 0.20 |
| Autoimmune disease | 0.053 | not measured consistently | |
| Obesity | 0.161 | 0.306 | <0.001 |
| 0 conditions | 0.474 | | |
| 1 condition | 0.274 | | |
| 2 conditions | 0.142 | | |
| >=3 conditions | 0.110 | | |
| Age 18-59 | 0.694 | 0.709 | 0.06 |
| Age 60-69 | 0.179 | 0.156 | 0.001 |
| Age 70+ | 0.128 | 0.135 | 0.22 |
| Male | 0.484 | 0.489 | 0.63 |
| Non-Hispanic white | 0.631 | 0.637 | 0.59 |
| Non-Hispanic black | 0.120 | 0.114 | 0.41 |
| Hispanic | 0.160 | 0.161 | 0.97 |
| Non-Hispanic other | 0.088 | 0.088 | >0.99 |
| <=12 yrs schooling | 0.375 | 0.350 | 0.01 |
| 13-15 yrs schooling | 0.271 | 0.307 | <0.001 |
| 16+ years schooling | 0.353 | 0.343 | 0.27 |

*UAS, but not NHIS, explicitly excludes skin cancer. Kidney disease is in the last year for NHIS.

© 2021 Schoeni RF et al. *JAMA Network Open.*

**eTable 4. OLS and unconditional median regressions of perceived percent chance of infection in next 3 months**

| Explanatory variables | OLS Regression 1 Beta/[95%CI] | OLS Regression 2 Beta/[95%CI] | Unconditional Median Regression 1 Beta/[95%CI] | Unconditional Median Regression 2 Beta/[95%CI] |
|---|---|---|---|---|
| Diabetes | 3.45* | | 4.94* | |
| | [0.75,6.14] | | [0.78,9.10] | |
| Cancer | 3.11 | | 1.90 | |
| | [-0.19,6.41] | | [-3.03,6.82] | |
| Heart disease | 3.30* | | 4.98 | |
| | [0.14,6.46] | | [-0.26,10.23] | |
| High blood pressure | -0.63 | | -1.09 | |
| | [-2.47,1.21] | | [-4.07,1.88] | |
| Asthma | 0.78 | | 2.03 | |
| | [-1.65,3.21] | | [-1.98,6.04] | |
| Chronic lung disease | 6.97** | | 8.38** | |
| | [2.78,11.15] | | [2.02,14.74] | |
| Kidney disease | 4.50 | | 2.97 | |
| | [-0.65,9.64] | | [-4.75,10.69] | |
| Autoimmune disease | 4.59** | | 7.21** | |
| | [1.42,7.77] | | [2.05,12.37] | |
| Obesity | 1.77 | | 4.78** | |
| | [-0.34,3.88] | | [1.30,8.26] | |
| Age 60-69 | -2.29* | -2.09* | -0.77 | -0.99 |
| | [-4.28,-0.30] | [-4.04,-0.14] | [-4.02,2.49] | [-4.21,2.22] |
| Age 70+ | -3.91** | -3.45** | -2.76 | -3.12 |
| | [-6.25,-1.57] | [-5.71,-1.19] | [-6.65,1.12] | [-6.83,0.60] |
| Male | -4.11*** | -4.22*** | -5.54*** | -5.96*** |
| | [-5.76,-2.45] | [-5.85,-2.59] | [-8.10,-2.97] | [-8.47,-3.44] |
| Non-Hispanic black | -2.93* | -3.32* | -3.99 | -4.61* |
| | [-5.74,-0.13] | [-6.09,-0.55] | [-8.33,0.35] | [-8.91,-0.32] |
| Hispanic | 1.31 | 1.26 | 1.69 | 1.69 |
| | [-1.47,4.09] | [-1.52,4.03] | [-2.44,5.82] | [-2.44,5.82] |
| Non-Hispanic other | -1.87 | -1.93 | -4.25 | -4.38 |
| | [-4.62,0.89] | [-4.69,0.84] | [-8.79,0.28] | [-8.92,0.17] |
| 13-15 yrs school | -1.28 | -1.32 | -3.39* | -3.32* |
| | [-3.33,0.77] | [-3.37,0.74] | [-6.54,-0.23] | [-6.47,-0.17] |
| 16+ yrs school | -2.30* | -2.44* | -1.98 | -1.99 |
| | [-4.25,-0.35] | [-4.38,-0.49] | [-5.03,1.07] | [-5.02,1.04] |
| 1 condition | | 1.00 | | 1.74 |
| | | [-0.92,2.92] | | [-1.34,4.82] |
| 2 conditions | | 2.99* | | 5.15** |
| | | [0.55,5.42] | | [1.30,8.99] |
| 3+ conditions | | 8.27*** | | 12.31*** |
| | | [5.53,11.02] | | [8.23,16.39] |
| Constant | 27.04*** | 27.17*** | | |
| | [25.07,29.00] | [25.13,29.20] | | |
| n | 5900 | 5900 | 5900 | 5900 |

Reference categories: 0 conditions, age 18-59, female, non-Hispanic white, <=12 years of schooling. * p<0.05, ** p<0.01, *** p<0.001

© 2021 Schoeni RF et al. *JAMA Network Open.*

**eTable 5. OLS and unconditional median regressions of perceived percent chance of hospitalization if infected**

| Explanatory variables | OLS Regression 1 Beta/[95%CI] | OLS Regression 2 Beta/[95%CI] | Unconditional Median Regression 1 Beta/[95%CI] | Unconditional Median Regression 2 Beta/[95%CI] |
|---|---|---|---|---|
| Diabetes | 8.46*** | | 4.18** | |
| | [5.22,11.71] | | [1.53,6.84] | |
| Cancer | 7.09*** | | 3.87* | |
| | [3.20,10.97] | | [0.83,6.92] | |
| Heart disease | 9.58*** | | 3.81* | |
| | [5.13,14.03] | | [0.38,7.24] | |
| High blood pressure | 1.58 | | 2.44** | |
| | [-0.47,3.64] | | [0.63,4.26] | |
| Asthma | 4.59** | | 3.59** | |
| | [1.80,7.37] | | [1.20,5.99] | |
| Chronic lung disease | 18.42*** | | 6.84** | |
| | [12.50,24.34] | | [2.37,11.32] | |
| Kidney disease | 5.47 | | 5.35 | |
| | [-0.69,11.64] | | [-0.22,10.92] | |
| Autoimmune disease | 7.93*** | | 4.63** | |
| | [3.73,12.14] | | [1.44,7.82] | |
| Obesity | 4.44*** | | 4.64*** | |
| | [2.10,6.79] | | [2.55,6.72] | |
| Age 60-69 | 4.64*** | 5.12*** | 4.54*** | 4.46*** |
| | [2.33,6.95] | [2.82,7.42] | [2.51,6.57] | [2.46,6.46] |
| Age 70+ | 9.79*** | 10.96*** | 7.58*** | 7.47*** |
| | [6.60,12.98] | [7.86,14.07] | [5.24,9.92] | [5.26,9.68] |
| Male | -0.77 | -0.69 | -1.16 | -1.31 |
| | [-2.51,0.97] | [-2.42,1.04] | [-2.72,0.41] | [-2.85,0.23] |
| Non-Hispanic black | 3.35* | 2.53 | 2.33 | 2.13 |
| | [0.27,6.42] | [-0.52,5.58] | [-0.28,4.95] | [-0.48,4.75] |
| Hispanic | 3.90** | 3.74** | 4.64*** | 4.62*** |
| | [1.22,6.58] | [1.03,6.45] | [2.20,7.08] | [2.19,7.05] |
| Non-Hispanic other | 3.53* | 3.56* | 2.88* | 2.87* |
| | [0.55,6.51] | [0.63,6.49] | [0.12,5.64] | [0.12,5.62] |
| 13-15 yrs school | -4.34*** | -4.56*** | -3.53*** | -3.52*** |
| | [-6.52,-2.16] | [-6.76,-2.36] | [-5.47,-1.59] | [-5.46,-1.59] |
| 16+ yrs school | -8.45*** | -8.90*** | -6.01*** | -6.04*** |
| | [-10.47,-6.42] | [-10.95,-6.85] | [-7.86,-4.17] | [-7.88,-4.19] |
| 1 condition | | 4.81*** | | 3.89*** |
| | | [2.79,6.83] | | [1.97,5.80] |
| 2 conditions | | 8.35*** | | 7.56*** |
| | | [5.69,11.01] | | [5.19,9.92] |
| 3+ conditions | | 24.23*** | | 14.06*** |
| | | [20.81,27.64] | | [11.75,16.36] |
| Constant | 18.71*** | 18.78*** | | |
| | [16.65,20.77] | [16.68,20.87] | | |
| n | 5899 | 5899 | 5899 | 5899 |

Reference categories: 0 conditions, age 18-59, female, non-Hispanic white, <=12 years of schooling.

* p<0.05, ** p<0.01, *** p<0.001

© 2021 Schoeni RF et al. *JAMA Network Open.*

## eTable 6. OLS and unconditional median regressions of perceived percent chance of death if infected

| Explanatory variables | OLS Regression 1 Beta/[95%CI] | OLS Regression 2 Beta/[95%CI] | Unconditional Median Regression 1 Beta/[95%CI] | Unconditional Median Regression 2 Beta/[95%CI] |
|---|---|---|---|---|
| Diabetes | 6.68*** | | 2.45*** | |
| | [3.64,9.72] | | [1.02,3.87] | |
| Cancer | 6.85*** | | 1.19 | |
| | [2.90,10.80] | | [-0.52,2.89] | |
| Heart disease | 11.19*** | | 2.97** | |
| | [6.64,15.74] | | [1.19,4.75] | |
| High blood pressure | 0.56 | | 1.32** | |
| | [-1.46,2.58] | | [0.35,2.28] | |
| Asthma | 3.02* | | 2.08** | |
| | [0.50,5.54] | | [0.82,3.34] | |
| Chronic lung disease | 20.33*** | | 4.14*** | |
| | [14.27,26.39] | | [1.76,6.51] | |
| Kidney disease | 4.76 | | 2.27 | |
| | [-1.66,11.18] | | [-0.67,5.20] | |
| Autoimmune disease | 6.86*** | | 2.84*** | |
| | [3.06,10.65] | | [1.16,4.52] | |
| Obesity | 2.15 | | 1.76** | |
| | [-0.03,4.34] | | [0.65,2.87] | |
| Age 60-69 | 4.42*** | 5.17*** | 2.46*** | 2.45*** |
| | [2.17,6.67] | [2.93,7.41] | [1.37,3.55] | [1.37,3.52] |
| Age 70+ | 7.94*** | 9.97*** | 5.02*** | 5.01*** |
| | [4.93,10.95] | [7.00,12.94] | [3.80,6.24] | [3.85,6.17] |
| Male | -1.99* | -1.73* | -1.42*** | -1.45*** |
| | [-3.63,-0.35] | [-3.38,-0.08] | [-2.24,-0.59] | [-2.27,-0.64] |
| Non-Hispanic black | 4.18** | 3.15* | 2.61*** | 2.45*** |
| | [1.18,7.18] | [0.19,6.10] | [1.19,4.03] | [1.03,3.87] |
| Hispanic | 4.91*** | 4.56*** | 3.67*** | 3.62*** |
| | [2.32,7.50] | [1.91,7.20] | [2.37,4.96] | [2.34,4.91] |
| Non-Hispanic other | 2.82* | 2.81* | 2.96*** | 2.96*** |
| | [0.10,5.55] | [0.14,5.47] | [1.48,4.44] | [1.49,4.44] |
| 13-15 yrs school | -5.18*** | -5.54*** | -1.69** | -1.72** |
| | [-7.26,-3.11] | [-7.65,-3.44] | [-2.72,-0.66] | [-2.75,-0.69] |
| 16+ yrs school | -9.61*** | -10.24*** | -3.87*** | -3.95*** |
| | [-11.56,-7.66] | [-12.22,-8.25] | [-4.86,-2.88] | [-4.93,-2.98] |
| 1 condition | | 4.15*** | | 2.25*** |
| | | [2.25,6.04] | | [1.24,3.26] |
| 2 conditions | | 5.58*** | | 3.82*** |
| | | [3.10,8.05] | | [2.54,5.11] |
| 3+ conditions | | 20.73*** | | 7.72*** |
| | | [17.34,24.12] | | [6.47,8.96] |
| Constant | 16.07*** | 16.01*** | | |
| | [14.06,18.08] | [13.99,18.04] | | |
| n | 5900 | 5900 | 5900 | 5900 |

Reference categories: 0 conditions, age 18-59, female, non-Hispanic white, <=12 years of schooling.

* p<0.05, ** p<0.01, *** p<0.001

© 2021 Schoeni RF et al. *JAMA Network Open.*

## eTable 7. Models of activities undertaken in the past 7 days

| Explanatory variable | Logit Bar/Club OR/[95%CI] | Logit Grocery /Pharmacy OR/[95%CI] | Logit Visit friend's home OR/[95%CI] | Logit Host visitors at home OR/[95%CI] | Logit Gathering of 10+ OR/[95%CI] | Logit Left home, non essential OR/[95%CI] | Logit Share towel/ utensil[a] OR/[95%CI] | Logit <6ft coresident[a] OR/[95%CI] | Logit <6ft non coresident OR/[95%CI] | Poisson # of activities[b] RR/[95%CI] |
|---|---|---|---|---|---|---|---|---|---|---|
| 1 condition | 1.16 | 1.24 | 1.08 | 1.05 | 0.99 | 0.86 | 1.00 | 1.57** | 1.20 | 1.03 |
| | [0.87,1.56] | [0.98,1.57] | [0.90,1.29] | [0.88,1.25] | [0.77,1.26] | [0.72,1.03] | [0.81,1.23] | [1.12,2.22] | [1.00,1.44] | [0.98,1.08] |
| 2 conditions | 0.74 | 1.17 | 0.93 | 1.03 | 0.90 | 0.73** | 0.92 | 1.40 | 1.05 | 0.97 |
| | [0.51,1.08] | [0.88,1.56] | [0.74,1.16] | [0.83,1.29] | [0.65,1.23] | [0.59,0.91] | [0.70,1.21] | [0.92,2.13] | [0.84,1.32] | [0.91,1.03] |
| 3+ conditions | 0.92 | 0.89 | 0.75* | 1.05 | 0.86 | 0.51*** | 0.81 | 1.02 | 0.91 | 0.91** |
| | [0.60,1.41] | [0.66,1.20] | [0.59,0.97] | [0.83,1.33] | [0.61,1.23] | [0.40,0.65] | [0.59,1.11] | [0.67,1.56] | [0.71,1.16] | [0.85,0.97] |
| Age 60-69 | 0.82 | 1.61*** | 0.84 | 0.84 | 0.86 | 0.54*** | 0.73** | 1.21 | 0.67*** | 0.91*** |
| | [0.60,1.12] | [1.24,2.10] | [0.70,1.02] | [0.70,1.01] | [0.66,1.13] | [0.45,0.65] | [0.58,0.93] | [0.85,1.74] | [0.56,0.81] | [0.87,0.96] |
| Age 70+ | 0.48*** | 1.03 | 0.64*** | 0.81* | 0.88 | 0.36*** | 0.97 | 1.41 | 0.57*** | 0.82*** |
| | [0.32,0.71] | [0.78,1.36] | [0.51,0.80] | [0.65,0.99] | [0.66,1.19] | [0.29,0.45] | [0.74,1.28] | [0.86,2.31] | [0.46,0.71] | [0.78,0.87] |
| Male | 1.67*** | 1.08 | 1.00 | 1.09 | 1.27* | 1.30*** | 0.88 | 1.03 | 1.19* | 1.07** |
| | [1.32,2.13] | [0.89,1.31] | [0.86,1.16] | [0.94,1.26] | [1.04,1.55] | [1.12,1.52] | [0.74,1.05] | [0.78,1.36] | [1.02,1.38] | [1.03,1.11] |
| Non-Hispanic black | 0.45** | 0.79 | 0.49*** | 0.57*** | 0.81 | 0.51*** | 0.27*** | 0.30*** | 0.45*** | 0.77*** |
| | [0.26,0.77] | [0.58,1.07] | [0.38,0.64] | [0.44,0.74] | [0.57,1.16] | [0.40,0.66] | [0.17,0.42] | [0.20,0.44] | [0.35,0.58] | [0.72,0.84] |
| Hispanic | 0.59* | 0.85 | 0.66*** | 0.57*** | 0.87 | 0.56*** | 0.76 | 0.34*** | 0.56*** | 0.83*** |
| | [0.38,0.92] | [0.64,1.15] | [0.52,0.84] | [0.45,0.73] | [0.62,1.22] | [0.44,0.71] | [0.57,1.01] | [0.24,0.48] | [0.44,0.71] | [0.77,0.89] |
| Non-Hispanic other | 0.45*** | 1.10 | 0.85 | 0.62*** | 0.55** | 0.50*** | 1.00 | 0.52** | 0.64*** | 0.84*** |
| | [0.28,0.72] | [0.78,1.54] | [0.66,1.11] | [0.48,0.82] | [0.37,0.83] | [0.38,0.65] | [0.74,1.36] | [0.33,0.81] | [0.49,0.83] | [0.78,0.91] |
| 13-15 yrs school | 1.38* | 1.06 | 1.10 | 0.90 | 0.87 | 1.04 | 1.16 | 1.32 | 1.12 | 1.01 |
| | [1.01,1.87] | [0.84,1.33] | [0.92,1.32] | [0.76,1.08] | [0.68,1.12] | [0.86,1.25] | [0.93,1.46] | [0.97,1.82] | [0.93,1.34] | [0.96,1.06] |
| 16+ yrs school | 1.21 | 1.14 | 0.89 | 0.82* | 0.99 | 0.96 | 1.41** | 1.62** | 1.14 | 0.99 |
| | [0.89,1.63] | [0.91,1.43] | [0.75,1.07] | [0.69,0.98] | [0.78,1.26] | [0.80,1.15] | [1.14,1.74] | [1.16,2.27] | [0.96,1.37] | [0.94,1.04] |
| Constant | 0.09*** | 3.71*** | 0.87 | 0.97 | 0.19*** | 2.24*** | 0.47*** | 8.01*** | 2.11*** | 3.38*** |
| | [0.07,0.13] | [2.96,4.65] | [0.73,1.04] | [0.82,1.16] | [0.15,0.25] | [1.86,2.70] | [0.38,0.58] | [5.81,11.03] | [1.75,2.55] | [3.22,3.55] |
| n | 5862 | 5859 | 5858 | 5869 | 5859 | 5841 | 4632 | 4652 | 5841 | 5744 |

Reference categories: 0 conditions, age 18-59, female, non-Hispanic white, <=12 years of schooling. OR=odds ratio. RR=rate ratio. * p<0.05, ** p<0.01, *** p<0.001. [a] Among those who live with someone else. [b] Excludes shared towel / utensils and <6ft from coresident because some people live alone.

© 2021 Schoeni RF et al. *JAMA Network Open.*

## eTable 8. Logistic models of mask wearing in the past 7 days

| | | Always wore mask past 7 days while at: | | | | | |
|---|---|---|---|---|---|---|---|
| Explanatory variables | Wore mask somewhere in past 7 days OR/[95%CI] | Bar/club OR/[95%CI] | Grocery/ pharmacy OR/[95%CI] | Visit friend's home OR/[95%CI] | Host visitor OR/[95%CI] | Gathering of 10+ OR/[95%CI] | <6ft noncoresident OR/[95%CI] |
| 1 condition | 1.59** | 1.61 | 1.06 | 1.27 | 1.04 | 1.58 | 1.01 |
| | [1.16,2.20] | [0.85,3.03] | [0.82,1.38] | [0.78,2.08] | [0.58,1.87] | [0.95,2.65] | [0.79,1.29] |
| 2 conditions | 1.63* | 2.33* | 1.40 | 1.79* | 1.99* | 2.20* | 1.48** |
| | [1.09,2.45] | [1.07,5.09] | [0.99,1.98] | [1.04,3.10] | [1.11,3.57] | [1.13,4.29] | [1.10,1.99] |
| 3+ conditions | 1.95** | 0.73 | 1.53* | 1.57 | 1.51 | 1.97 | 1.06 |
| | [1.19,3.20] | [0.31,1.76] | [1.04,2.27] | [0.80,3.07] | [0.79,2.91] | [0.97,3.99] | [0.74,1.50] |
| Age 60-69 | 2.28*** | 2.53** | 1.87*** | 1.28 | 1.23 | 3.30*** | 1.15 |
| | [1.55,3.36] | [1.28,5.00] | [1.38,2.55] | [0.78,2.10] | [0.74,2.07] | [1.93,5.64] | [0.88,1.50] |
| Age 70+ | 2.31*** | 3.11** | 2.00*** | 1.44 | 1.33 | 3.48*** | 1.16 |
| | [1.45,3.67] | [1.40,6.89] | [1.40,2.85] | [0.78,2.68] | [0.73,2.42] | [1.91,6.34] | [0.86,1.58] |
| Male | 0.76* | 0.65 | 0.73** | 0.75 | 0.68 | 1.27 | 0.88 |
| | [0.59,0.98] | [0.38,1.14] | [0.59,0.91] | [0.50,1.14] | [0.43,1.06] | [0.81,1.97] | [0.72,1.09] |
| Non-Hispanic black | 2.28** | 1.77 | 1.52* | 4.91*** | 6.25*** | 3.36** | 2.74*** |
| | [1.36,3.81] | [0.64,4.90] | [1.01,2.30] | [2.79,8.66] | [3.59,10.86] | [1.55,7.28] | [1.95,3.85] |
| Hispanic | 1.48 | 1.51 | 1.32 | 1.85* | 1.81 | 1.71 | 2.29*** |
| | [0.98,2.23] | [0.59,3.87] | [0.91,1.90] | [1.00,3.43] | [0.86,3.79] | [0.90,3.28] | [1.66,3.16] |
| Non-Hispanic other | 2.26** | 0.38 | 0.94 | 2.03* | 2.60* | 2.70* | 1.61** |
| | [1.38,3.70] | [0.14,1.05] | [0.65,1.38] | [1.03,3.98] | [1.18,5.73] | [1.18,6.21] | [1.13,2.29] |
| 13-15 yrs school | 1.23 | 1.48 | 1.58*** | 1.37 | 1.14 | 1.19 | 1.29 |
| | [0.92,1.63] | [0.73,3.01] | [1.24,2.03] | [0.82,2.27] | [0.66,1.94] | [0.68,2.08] | [0.99,1.67] |
| 16+ yrs school | 2.56*** | 1.54 | 3.51*** | 1.25 | 1.18 | 2.63*** | 1.22 |
| | [1.85,3.54] | [0.79,3.00] | [2.67,4.63] | [0.76,2.06] | [0.71,1.98] | [1.52,4.55] | [0.95,1.57] |
| Constant | 4.00*** | 0.19*** | 2.38*** | 0.05*** | 0.04*** | 0.08*** | 0.24*** |
| | [3.01,5.31] | [0.09,0.39] | [1.84,3.07] | [0.03,0.08] | [0.02,0.08] | [0.04,0.15] | [0.19,0.32] |
| n | 5901 | 522 | 4857 | 2287 | 2414 | 801 | 3704 |

Reference categories: 0 conditions, age 18-59, female, non-Hispanic white, <=12 years of schooling. * p<0.05, ** p<0.01, *** p<0.001. OR=odds ratio.

© 2021 Schoeni RF et al. *JAMA Network Open.*

## eTable 9. Logistic models of whether agree or strongly agree about various aspects of wearing a mask

| Explanatory variables | Keeps me safe OR/[95%CI] | Keeps others safe OR/[95%CI] | Is dangerous OR/[95%CI] | Is political statement OR/[95%CI] | Not needed if not infected OR/[95%CI] | Not needed if others healthy OR/[95%CI] | Is threatening to others OR/[95%CI] | Not needed if keep distance OR/[95%CI] | No one can force me to wear OR/[95%CI] | Can't afford OR/[95%CI] | COVID-19 not serious OR/[95%CI] | Is un-comfortabl e OR/[95%CI] |
|---|---|---|---|---|---|---|---|---|---|---|---|---|
| 1 condition | 1.25* | 1.32** | 0.67* | 0.71* | 0.56** | 0.55*** | 1.04 | 0.83 | 0.79* | 1.16 | 0.43*** | 0.89 |
| | [1.04,1.51] | [1.08,1.61] | [0.49,0.93] | [0.54,0.93] | [0.39,0.79] | [0.42,0.72] | [0.69,1.56] | [0.62,1.11] | [0.63,0.99] | [0.57,2.35] | [0.28,0.66] | [0.71,1.11] |
| 2 conditions | 1.24 | 1.37* | 0.87 | 0.71* | 0.61* | 0.65* | 1.08 | 0.74 | 0.85 | 1.17 | 0.45** | 0.75 |
| | [0.97,1.58] | [1.06,1.77] | [0.60,1.26] | [0.50,1.00] | [0.39,0.95] | [0.46,0.91] | [0.65,1.78] | [0.51,1.06] | [0.64,1.13] | [0.45,3.05] | [0.26,0.78] | [0.55,1.02] |
| 3+ conditions | 1.69*** | 1.65*** | 0.76 | 0.52** | 0.27*** | 0.64* | 0.56 | 0.59* | 0.70* | 0.94 | 0.29*** | 0.81 |
| | [1.29,2.22] | [1.23,2.22] | [0.49,1.17] | [0.35,0.77] | [0.15,0.49] | [0.43,0.94] | [0.29,1.08] | [0.38,0.90] | [0.51,0.95] | [0.30,2.95] | [0.16,0.52] | [0.59,1.13] |
| Age 60-69 | 1.90*** | 1.97*** | 0.63* | 0.78 | 0.51** | 0.70* | 0.57* | 0.48*** | 0.73** | 0.05*** | 0.41** | 0.60*** |
| | [1.54,2.34] | [1.57,2.47] | [0.44,0.91] | [0.58,1.05] | [0.33,0.79] | [0.52,0.94] | [0.34,0.95] | [0.34,0.70] | [0.57,0.92] | [0.01,0.21] | [0.24,0.71] | [0.47,0.78] |
| Age 70+ | 1.82*** | 1.76*** | 0.48*** | 0.60** | 0.46** | 0.61** | 0.36** | 0.42*** | 0.61*** | 0.47 | 0.36*** | 0.55*** |
| | [1.43,2.31] | [1.36,2.28] | [0.31,0.72] | [0.43,0.85] | [0.27,0.78] | [0.43,0.87] | [0.20,0.67] | [0.28,0.63] | [0.46,0.81] | [0.16,1.44] | [0.20,0.65] | [0.41,0.73] |
| Male | 0.80** | 0.82* | 1.16 | 1.52*** | 1.60** | 1.48*** | 1.62** | 1.10 | 1.49*** | 0.9 | 1.84*** | 1.12 |
| | [0.68,0.93] | [0.70,0.97] | [0.91,1.49] | [1.22,1.90] | [1.21,2.11] | [1.18,1.84] | [1.14,2.30] | [0.87,1.40] | [1.24,1.78] | [0.49,1.66] | [1.34,2.52] | [0.93,1.34] |
| Non-Hispanic black | 2.55*** | 1.67*** | 0.33*** | 0.27*** | 0.15*** | 0.25*** | 1.55 | 0.32*** | 0.32*** | 1.71 | 0.19*** | 0.43*** |
| | [1.88,3.45] | [1.24,2.26] | [0.19,0.60] | [0.15,0.49] | [0.06,0.39] | [0.14,0.43] | [0.92,2.63] | [0.18,0.57] | [0.22,0.48] | [0.73,4.01] | [0.07,0.51] | [0.30,0.63] |
| Hispanic | 1.98*** | 1.56** | 0.77 | 0.52** | 0.46** | 0.63* | 1.22 | 0.66* | 0.45*** | 1.93 | 0.42** | 0.94 |
| | [1.52,2.59] | [1.18,2.05] | [0.51,1.15] | [0.34,0.78] | [0.27,0.79] | [0.43,0.92] | [0.73,2.03] | [0.44,0.97] | [0.32,0.63] | [0.89,4.16] | [0.23,0.77] | [0.70,1.25] |
| Non-Hispanic other | 1.54** | 1.43* | 0.50** | 0.48** | 0.56* | 0.52** | 1.06 | 0.45*** | 0.51*** | 1.37 | 0.73 | 0.75 |
| | [1.15,2.06] | [1.05,1.96] | [0.31,0.81] | [0.31,0.76] | [0.33,0.93] | [0.35,0.79] | [0.58,1.94] | [0.29,0.73] | [0.36,0.72] | [0.49,3.81] | [0.43,1.25] | [0.53,1.07] |
| 13-15 yrs school | 1.19 | 1.40*** | 0.76 | 0.96 | 0.99 | 1.03 | 0.95 | 0.98 | 0.93 | 0.60 | 0.77 | 0.71** |
| | [0.98,1.43] | [1.15,1.70] | [0.57,1.01] | [0.73,1.25] | [0.72,1.38] | [0.79,1.35] | [0.63,1.45] | [0.73,1.31] | [0.74,1.15] | [0.30,1.20] | [0.53,1.13] | [0.58,0.89] |
| 16+ yrs school | 2.32*** | 2.87*** | 0.44*** | 0.65** | 0.50*** | 0.79 | 0.76 | 0.83 | 0.60*** | 0.38* | 0.49*** | 0.44*** |
| | [1.92,2.82] | [2.33,3.53] | [0.32,0.60] | [0.50,0.85] | [0.35,0.72] | [0.60,1.03] | [0.50,1.17] | [0.63,1.10] | [0.48,0.75] | [0.17,0.85] | [0.33,0.71] | [0.35,0.55] |
| Constant | 0.94 | 1.17 | 0.22*** | 0.22*** | 0.16*** | 0.22*** | 0.05*** | 0.21*** | 0.39*** | 0.03*** | 0.14*** | 0.45*** |
| | [0.78,1.13] | [0.96,1.41] | [0.17,0.29] | [0.17,0.28] | [0.11,0.21] | [0.17,0.28] | [0.03,0.07] | [0.16,0.27] | [0.31,0.49] | [0.01,0.05] | [0.09,0.20] | [0.37,0.56] |
| n | 5897 | 5903 | 5902 | 5902 | 5900 | 5905 | 5904 | 5903 | 5901 | 5902 | 5903 | 5903 |

Reference categories: 0 conditions, age 18-59, female, non-Hispanic white, <=12 years of schooling.* p<0.05, ** p<0.01, *** p<0.001

© 2021 Schoeni RF et al. *JAMA Network Open.*

**eTable 10. Adjusted proportion wearing a mask in the past 7 days, by CDC risk factor [95% CI], with controls for opinions about masks**

| | CDC risk factor | | | | | | |
|---|---|---|---|---|---|---|---|
| | Number of conditions | | | | Age | | |
| | 0 | 1 | 2 | ≥3 | 18-59 | 60-69 | ≥70 |
| Wore a mask somewhere in past 7 days | | | | | | | |
| | 0.895 | 0.911 | 0.911 | 0.909 | 0.896 | 0.924 | 0.922 |
| | [0.880,0.910] | [0.892,0.930] | [0.887,0.935] | [0.875,0.943] | [0.883,0.909] | [0.904,0.945] | [0.896,0.948] |
| | | p=0.17 | p=0.28 | p=0.48 | | p=0.02 | p=0.10 |
| Always wore a mask in past 7 days while at: | | | | | | | |
| Bar/Club | 0.238 | 0.293 | 0.388 | 0.151 | 0.225 | 0.344 | 0.398 |
| | [0.168,0.308] | [0.206,0.380] | [0.250,0.526] | [0.055,0.247] | [0.167,0.282] | [0.231,0.457] | [0.250,0.545] |
| | | p=0.31 | p=0.04 | p=0.19 | | p=0.05 | p=0.02 |
| Grocery/Pharmacy | 0.830 | 0.819 | 0.846 | 0.835 | 0.818 | 0.856 | 0.859 |
| | [0.808,0.851] | [0.793,0.845] | [0.813,0.878] | [0.794,0.875] | [0.800,0.836] | [0.826,0.885] | [0.826,0.891] |
| | | p=0.51 | p=0.42 | p=0.82 | | p=0.04 | p=0.04 |
| Visiting friend's home | 0.081 | 0.090 | 0.127 | 0.100 | 0.088 | 0.098 | 0.108 |
| | [0.058,0.104] | [0.062,0.118] | [0.081,0.173] | [0.050,0.149] | [0.069,0.108] | [0.064,0.132] | [0.060,0.157] |
| | | p=0.62 | p=0.06 | p=0.48 | | p=0.63 | p=0.42 |
| Hosting visitors | 0.066 | 0.064 | 0.114 | 0.089 | 0.073 | 0.079 | 0.082 |
| | [0.044,0.087] | [0.039,0.089] | [0.071,0.158] | [0.048,0.130] | [0.055,0.092] | [0.050,0.109] | [0.045,0.119] |
| | | p=0.93 | p=0.03 | p=0.30 | | p=0.72 | p=0.67 |
| Gathering of 10+ | 0.251 | 0.291 | 0.379 | 0.306 | 0.234 | 0.405 | 0.395 |
| | [0.195,0.307] | [0.226,0.356] | [0.276,0.482] | [0.196,0.416] | [0.185,0.282] | [0.326,0.484] | [0.296,0.494] |
| | | p=0.31 | p=0.02 | p=0.37 | | p<0.001 | p=0.003 |
| <6ft of noncoresident | 0.281 | 0.261 | 0.351 | 0.262 | 0.286 | 0.276 | 0.274 |
| | [0.251,0.311] | [0.226,0.295] | [0.299,0.403] | [0.208,0.317] | [0.261,0.311] | [0.233,0.320] | [0.224,0.324] |
| | | p=0.38 | p=0.02 | p=0.57 | | p=0.71 | p=0.68 |

Adjusted estimates based on models controlling for number of conditions, age, gender, race-ethnicity, and education, as well as 12 opinions about mask wearing. p-values are associated with the coefficients on number of conditions relative to 0 conditions and age relative to 18-59 years old in these multivariable models.

© 2021 Schoeni RF et al. *JAMA Network Open.*
